# WhatisKT wiki: a case study of a platform for knowledge translation terms and definitions — descriptive analysis

**DOI:** 10.1186/1748-5908-8-13

**Published:** 2013-01-24

**Authors:** Kathleen Ann McKibbon, Cynthia Lokker, Arun Keepanasseril, Heather Colquhoun, Robert Brian Haynes, Nancy L Wilczynski

**Affiliations:** 1 Health Information Research Unit, Dept of Clinical Epidemiology and Biostatistics, McMaster University, CRL Building, 1280 Main Street West, Hamilton, ON L8S 4K1, Canada; 2 Centre for Practice-Changing Research (CPRC), Ottawa Hospital Research Institute. Ottawa Hospital – General Campus, 502 Smyth Road, Box 711, Ottawa, ON K1H 8L6, Canada

**Keywords:** Diffusion of innovations, Research, Knowledge translation, Implementation science

## Abstract

**Background:**

More than a hundred terms, often with unclear definitions and varying emphases, are used by health research and practice communities across the world who are interested in getting the best possible evidence applied (*e.g.*, knowledge translation, implementation science, diffusion of innovations, and technology transfer). This makes finding published evidence difficult and can result in reduced, misinterpreted, or challenging interactions among professionals. Open dialogue and interaction among various professionals is needed to achieve consolidation of vocabulary. We use case report methods to describe how we sought to build an online tool to present the range of terms and facilitate the dialogue process across groups and disciplines interested in harnessing research evidence for healthcare.

**Methods:**

We used a wiki platform from Wikispaces to present the problem of terminology and make a case and opportunity for collaboration on usage. Wikis are web sites where communities of users can collaborate online to build content and discuss progress. We gathered terms related to getting research into practice, sought published definitions, and posted these on the wiki (WhatisKT http://whatiskt.wikispaces.com/). We built the wiki in mid-2008 and promoted it through various groups and publications. This report describes the content of the site, our promotion efforts, use of the site, and how the site was used for collaboration up to the end of 2011.

**Results:**

The WhatisKT wiki site now includes more than 120 pages. Traffic to the site has increased substantially from an average of 200 monthly visits in 2008 to 1700 in 2011. Visitors from 143 countries viewed the wiki in 2011, compared with 12 countries in 2008. However, most use has been limited to short term accesses of about 40 seconds per visit, and discussion of consolidation and solidifying terminology is conspicuously absent.

**Conclusions:**

Although considerable interest exists in the terms and definitions related to getting research into practice based on increasing numbers of accesses, use of the WhatisKT wiki site for anything beyond quick lookups was minimal. Additional efforts must be directed towards increasing the level of interaction among the members of the site to encourage collaboration on term use.

## Background

The concept of using research findings to improve knowledge and practice behavior is broad and rapidly evolving, with many names such as knowledge translation (KT), dissemination, uptake, and implementation science. For this article, we have chosen to use the term KT. Regardless of what term is used, the concept generally encompasses the processes aimed at converting scientific knowledge to socially beneficial actions, often through behavior change of various stakeholders and actions of decision and policy makers. The scope of KT includes ‘knowledge dissemination, communication, technology transfer, ethical context, knowledge management, knowledge utilization, two-way exchange between researchers and those who apply knowledge, implementation research, technology assessment, synthesis of results within a global context, development of consensus guidelines and more’ [1]. The primary purpose of KT is to ‘address the gap between what is known from research and implementation of this knowledge by key stakeholders’ [2]. The specific design of these KT activities is dependent on the type of research knowledge to be transmitted and the target audience.

The terminology in the field is wide ranging, often with unclear definitions and varying emphases on KT aspects and their importance. With more than 100 terms in use across a wide spectrum of disciplines, navigating through KT terminology can be challenging for researchers, practitioners, and administrators [3]. Although divergent definitions can enrich a new field by adding dimensions to a given term or concept, lack of accepted semantics may prevent or decrease the quality of interactions among professionals, especially when the interactions cross national and regional boundaries or disciplines. Unclear definitions and inconsistent use of terminology also make finding published evidence challenging, leading to duplication of effort, knowledge gaps, and challenges in the production of knowledge summaries. The fledgling field of KT needs an open dialogue and exchange of ideas among stakeholders to understand and potentially consolidate its related vocabulary. Several online tools can facilitate this process.

One such tool is a wiki, designed to allow collaboration and consensus building by encouraging online dialogue and allowing modification of text by a defined user group. The concept of wikis became popular after the launch of Wikipedia in 2001 [4]. Many groups have used wikis, including businesses [5], all levels of education (kindergarten to graduate school) [6], civil liberties unions [7], and medical groups [8]. Wikis have been used to prepare research publications [9], design research [10], build eResearch Infrastructure [11], improve teaching and learning for nursing research teams [12], provide reminders to promote best practices in trauma care [13], develop software and learning [14], enable professional development [15], and produce project management tools [16]. Wikis can be open to everyone with no restrictions on who can modify content, open to all for access but only allowing modification by members, or can be open only to a predefined community.

Blogs, discussion boards, and wikis have features that provide these collaborative traits. Blogs and discussion boards are more personal and provide static content that is often presented in chronological order. We chose to use a wiki, designed to allow collaboration and consensus building by encouraging online dialogue and allowing modification of text by a defined user group. Wikis excel when a community of users wants to build a shared repository of knowledge [17]. Wikis have advantages over other collaboration tools and methods. They allow creation and maintenance of documents in dynamic and collaborative ways while keeping all versions of the documents (versioning) produced by multiple authors [14,18,19]. Wikis also allow incremental development of knowledge bases [20] and promote transparency in knowledge creation [16]. Selected information can be placed online to facilitate discovery and discussion [21].

Wikis are extremely easy to use and edit, requiring little to no technical skill [18]. They are often inexpensive to start and do not need in-house computer support, design experts, or trainers, making them low-cost and low-maintenance.

Collaborative innovation resulting from use of wikis has the potential to improve the quality of work and reports. KT researchers and practitioners could benefit from collaborative work to understand and build consensus, as well as to communicate the findings of KT research and evaluation. Therefore, we felt that use of a wiki for the broad, multinational network of KT researchers could facilitate resolution and understanding of our fields’ terminology challenges. Other groups have used wikis for their vocabulary work. The US Environmental Protection Agency, through their Terminology Services group, uses internal wiki-based platforms for collaborative vocabulary building. They have developed a suite of tools to help users understand the Agency and make their information “findable” [22]. Researchers at Stanford Center for Biomedical Informatics Research have developed web-based tools that allow ontology building by a dispersed community [23]. Researchers at the US National Cancer Institute and the Kaiser Permanente Colorado Cancer Communication Research Center have built a wiki for their community of users based on outcome measures and vocabulary related to dissemination and implementation [24].

The objectives of this article are to describe the development of the WhatisKT wiki, how we sought to disseminate the wiki, how it has been used (2008–2011) and how successful we have been. This article is a case report of our experience and findings.

## Methods

The WhatisKT wiki was created on the wikispaces platform. Wikispaces was selected because it offered easy editing, reasonable rates, universal access not tied to an individual institution, and familiarity — it is one of the largest of the wiki platforms. As of March 2011, Wikispaces had over 7.3 million registered users, more than 3.1 million all-time wikisites, and more than 105 million pages were viewed per month [9]. We use Google analytics to supplement the analytical data we receive from wikispaces.

User participation is the cornerstone of any wiki. The WhatisKT wiki offers several avenues for participation and collaboration to the users. We have incorporated design features keeping in mind the wide range of potential users of the wiki. For example, participants can join the WhatisKT wiki and then edit, review, comment, or add content. Members also have the option of joining an existing discussion or starting a new thread by posting in the discussion page.

### Initial building of WhatisKT wiki

Our first goal in the project was to collect terms related to the practice and theory of KT and their definitions and post them on one site. We started with the list of KT terms provided by Graham and colleagues [2]. Using these terms, we sought definitions for each KT term systematically by searching journals, books, and the Internet. Journals and books were searched using standard information retrieval methods and iterative cycling using newly-identified terms. In Google we used three search strings with each of our identified KT terms:

1.‘term,’ *e.g.*, ‘knowledge translation’

2. define: ‘term,’ *e.g.*, define: ‘knowledge translation’

3. ‘term’ definition, *e.g.*, ‘knowledge translation’ definition

We reviewed the links for the first 20 search results from each of the search strings for each term. If a definition was found, the URL and the definition were captured in a database. Any cited references in the definition or organizations that authored the definition also were collected, as was the date of last update for the website, if available. Duplicate definitions were tracked through their sources. We determined the discipline from which the definition came (*e.g.*, psychology, education) and the specific context of the definition, website (*e.g.*, research training), or both. Definitions came from a wide variety of disciplines, from nursing and medicine to sociology, marketing, and organizational behavior. We also contacted authors, librarians, and content experts, and searched the relevant KT literature and technical reports. The process of searching and compiling terms and their definitions was iterative and completed over the course of six months, with periodic updating and addition of new terms. In late 2011, 107 terms are included on the wiki site. The terms are divided into ‘KT terms’ for core terms and ‘other terms and concepts related to KT.’ Core terms were those that had features of both the knowledge and moving that knowledge into practice or use. Other terms were those that were more peripheral, such as communication, information, and evaluation.

### Links to other KT-related sites and features

The home page also features links to such trusted sources of the high quality literature of KT as KT + (http://plus.mcmaster.ca/KT/Default.aspx) and other sites we felt would be potentially valuable to those interested in the study or application of KT. WhatisKT periodically hosts polls and surveys on various aspects of KT, and provides access to the results of these polls. A user also has the option of receiving email notifications of updates for selected pages or for the entire wiki.

### Proposed ‘ideal’ definitions framework

Because we were interested in providing a forum for communication around definitions and increased awareness of how individuals and groups are using KT terms, we started a process whereby we proposed a single definition that we felt could be embraced by KT researchers and users. We hoped that readers would dialogue using the wiki format to start the process of coming to a common understanding of KT-related terms.

In spring 2011, we chose the terms that were viewed most frequently and selected 13 (Table 1). We used the broad and very commonly used definition of KT provided by the Canadian Institutes for Health Research (CIHR) [1] to determine the concepts or issues that are components of KT. Authors McKibbon (KAM) and Keepanasseril (AK) ultimately identified 12 concepts within the CIHR definition [1] that were integral to KT: the knowledge to be transferred or translated; the need for that knowledge; the intervention for translation; how the information is to be applied; who is to receive the knowledge; who has the knowledge; what are important features of the context or setting; what skills are needed; what tools are needed to implement the knowledge; what are the barriers and facilitators to implementation; what are the outcomes and how are they measured; and how is the project going to be evaluated and monitored.

**Table 1:** Percentage of visits for 13 terms with proposed definitions before and after inclusion of a preferred definition

**Term**	**Number (%) of term accesses December 2010 to May 2011 (6 months) (total accesses = 7840)**	**Number (%) of term accesses July 2011 to December 2011 (6 months) (total accesses = 14462)**	**Difference in percentage accessed (%)**
Research utilization	784 (10.0)	1,793 (12.4)	2.4
Diffusion of innovation	207 (2.64)	332 (2.31)	−0.49
Innovation adoption	202 (2.85)	917 (6.34)	0.72
Implementation science	58 (5.69)	531 (3.67)	0.68
Dissemination	186 (2.37)	492 (3.49)	1.06
Innovation adaptation	768 (0.98)	189 (1.31)	0.33
Knowledge communication	24 (0.30)	71 (0.49)	0.19
Diffusion	154 (1.96)	298 (2.06)	0.08
Capacity building	205 (2.61)	496 (3.43)	0.82
Knowledge dissemination	375 (4.78)	739 (5.11)	0.32
Knowledge cycle	65 (0.83)	101 (0.70)	−0.13
Knowledge mobilization	67 (0.86)	234 (1.62)	0.76
Knowledge transformation	11 (0.14)	27 (0.19)	0.05
Total accesses for the 13 terms	2,830 (36.1)	6,190 (42.8)	6.7

KAM and AK independently analyzed the definitions for each of the 13 high-access terms (Table 1) to determine how many of the above KT concepts were included in each definition as a measure of what components of KT each definition covered. Although we acknowledge that few terms related to KT would include all of these components, the definitions for each of the 13 terms with the most concepts were further reviewed for clarity, comprehensiveness, reputation of source, and breadth of coverage. One preferred published definition for each of the 13 terms was selected based on consensus, taking into consideration inclusion of important concepts of KT and representing what the majority of the definitions for that term included. The selected definition was moved to the top of the list of definitions on the wiki page for that term and highlighted. We made note in the wiki that we were suggesting the use of that definition over the others. We asked our readers to comment on our choice, and if they did not agree, to make suggestions for a better selection of definition. To determine what our readers were reviewing and if they were commenting on our definitions, we collected data on the degree to which these 13 terms were accessed.

### Dissemination

In June 2008, we invited colleagues and fellow KT researchers to join the WhatisKT wiki as members through several emailings. These mailings went out to colleagues of the authors and two mailing lists from KT Canada. We encouraged others to also mail out notice of our wiki. We urged the use of WhatisKT wiki in multiple publications [3,25,26]. We notified directors of KT courses of the wiki, including four annual KT Canada Summer Institutes (2009–2012). We also presented sessions at conferences and workshops, and presented webinars introducing the vocabulary challenges of KT and encouraging the use of the wiki for information and also consensus building. The KT Canada KT Tools webpage links to WhatisKT (http://ktclearinghouse.ca/tools/uncategorized), as do other KT websites, such as Implementation Central (http://www.implementationcentral.com/index.html), KT + (http://plus.mcmaster.ca/kt/Default.aspx), and the Alberta Innovates Health Solutions. (http://www.aihealthsolutions.ca/rtna/links.php).

### Data analysis

Using data collected by wikispaces and Google analytics, we descriptively assessed usage and traffic trends for 2008 to the end of 2011. We also determined how the users came to the WhatisKT wiki by using internet statistics sources and searching withGoogle and other search engines, such as Bing, Dogpile, and Altavista. Wiki traffic results are presented as monthly averages with the years broken down into six-month divisions for 2008 to 2011. Google analytics data are not available from June 29, 2010 to February 18, 2011 because of a lapse in our subscription.

## Results

As of the end of 2011, the WhatisKT wiki has 107 pages, one for each KT term. Each term related to KT includes at least one published definition, with most having multiple definitions. The number of visits to the wiki has increased substantially from 2008, when we had 200 unique visitors (unduplicated visitors, counted only once, in a specific timeframe) per month to more than 1,700 unique visitors per month in the last half of 2011 (Table 2). The percentage of returning visitors is low but has also been increasing from less than 3% in 2008 to 6.5% for all of 2011 (Table 2). With only 72 registered users, most use is from non-members.

**Table 2:** Average number of visitors per month to WhatisKT wiki for 6 month intervals

**Time Period**	**Average visits per month**	**Average unique visitors per month**	**Average number of pages viewed per month**	**Returning visitor (%)**
July-Dec. 2008	201	196	298	2.7
Jan-June 2009	304	292	423	4.0
July-Dec. 2009	580	545	871	6.5
Jan-June 2010	535	505	652	5.6
July-Dec. 2010	Missing data	Missing data	Missing data	Missing data
Jan-Jun 2011	1104	1004	1831	8.5
July-Dec 2011	1835	1720	2410	6.5

We were hoping for interaction among users to include commenting on terms and suggested definitions — the main goal of the project. Several people suggested additional terms throughout the process. However, the most discouraging fact is that no one outside of our working group (up to 10 people at various times) used the WhatisKT wiki to comment on content, discuss proposed definitions, or to take part in other activities such as discussion groups.

The wiki garnered world-wide use with visitors from 12 countries in 2008 increasing to 143 countries in 2011. Table 3 lists the location of visitors who accessed the wiki from 2008 to 2011. Canada had the highest proportion of visitors for the first two years; the proportion of US visitors has remained relatively unchanged since 2009, at approximately one-third of all visitors. All other countries showed substantial increases in use, especially the United Kingdom and India. Visitors from China began accessing the wiki in 2011. Australia and New Zealand had visitors in all years.

**Table 3:** Percentage of visitors to WhatisKT by country for 2008-2011

**Year**	**Canada**	**US**	**UK**	**India**	**Australia**	**Others**
2008	82.3	13.5	0.6	0.0	1.7	2.7
2009	43.2	34.7	7.6	0.7	1.4	7.0
2010	26.1	32.8	12.4	4.6	3.4	9
2011	15.5	34.3	8.3	8.2	3.9	33

Average time spent by a visitor in the wiki was 16 seconds in the first half of 2009 and 57 seconds in the corresponding period in 2011 (Table 4). The bounce rate (percentage of users who only visit one webpage before leaving the site) ranged from 88% to 97% (Table 3).

**Table 4:** Average time spent on WhatisKT wiki and bounce rate for 6 month intervals

**Time Period**	**Average time per visit in seconds**	**Average number of pages per visit**	**Average time per page in seconds**	Bounce rate* (%)
July-Dec. 2008	22	1.5	15	97
Jan-June 2009	16	1.4	11	96
July-Dec. 2009	37	1.5	16	94
Jan-June 2010	23	1.2	19	93
July-Dec. 2010	Missing data†	Missing data†	Missing data†	Missing data†
Jan-June 2011	57	1.6	36	88
July-Dec. 2011	30	1.3	25	92

*“Bounce rate is the percentage of single-page visits or visits in which the person left the site from the entrance or landing page”. (http://www.google.com/support/analytics/bin/answer.py?answer=81986).

†Google analytics data for this period was not recorded due to lapsed subscription.

Table 5 lists the top 10 KT terms according to the number of times they were accessed during the four years of observation. Although the ranking of terms is somewhat different across time periods, the terms accessed remained similar, especially for those accessed most frequently. We have included the number of accesses for 2011 to show the spread of number of visits. In 2011, ‘research utilization’ was viewed 4,030 times, while ‘diffusion of innovation’ was viewed 950 times. The least viewed terms for 2011 were ‘routinization’ (63 times), ‘product adoption and utilization’ (81 times), and ‘potentially better products’ (81 times). Eighteen other terms were viewed less than 100 times.

**Table 5:** Ten most frequently viewed KT term pages by year for the WhatisKT wiki

**Rank**	**Top pages-2008**	**Top pages −2009**	**Top pages −2010**	Top pages −2011 (number of accesses)**
1	Applied Dissemination	Research Utilization	Research Utilization	Research Utilization (4297)
2	Knowledge Translation	Knowledge Translation	Innovation Adoption	Innovation Adoption (2707)
3	Capacity Building	Implementation Science	Implementation Science	Knowledge Cycle (2457)
4	Applied Health Research	Innovation Adoption	Knowledge Dissemination	Dissemination (1546)
5	Knowledge Brokering	Implementation Research	Knowledge Synthesis	Implementation Science (1259)
6	Diffusion of Innovation	Dissemination	Dissemination	Knowledge Dissemination (1221)
7	Integrated KT	Knowledge Management	Knowledge Translation	Knowledge Transfer (1206)
8	Knowledge Management	Knowledge Dissemination	Capacity Building	Knowledge Mobilization (1129)
9	Knowledge Mobilization	Knowledge Transfer	Diffusion of Innovation	Capacity Building (1227)
10	Change Implementation	Action Research	Implementation Research	Diffusion of Innovation (1035)

Table 1 shows the changes in the proportion of page views following changes in content. After implementing, the 13 enhanced definitions represented a greater proportion of the pages viewed after the terms were flagged as having a proposed definition than compared to before term flagging (P < 0.001for testing of proportions comparing accesses in the six months before and six months after implementation of the definitions). ‘Research utilization’ received the most page views both before and after the edits adding in the preferred definitions.

In 2008 and 2009, most visitors reached the site directly by typing or linking to the site URL in their browser address bar (86% and 72%, respectively). The rate of direct access has decreased and is now stabilized at approximately 30% (Table 6). Wikispaces, the hosting platform for the wiki, initially directed a few visitors, and this rate has steadily decreased. The proportion of people reaching the wiki through Google’s internet search engine has risen consistently over the years, from 2.4% in 2008 to 20% in 2009, 59% in 2010, and 67% in 2011. Google searches list WhatisKT as one of the first sites when Google is asked for ‘definitions’ or ‘define’ with any of the KT terms.

**Table 6:** Top 3 access points that people used to access WhatisKT by year

**Year**	**Source of access to WhatisKT wiki**	**% of traffic from source**
2008	Direct link	86
	Wikispaces.com	4.1
	Google	2.4
2009	Direct link	72
	Google	20
	Wikispaces.com	1.2
2010	Direct link	30
	Google	59
	Wikispaces.com	1.2
2011	Direct link	20
	Google	68
	Bing	2.1

## Discussion

The number of people using the WhatisKT wiki has steadily increased from 2008 to 2011, with average monthly visits increasing over 500% during this period. Similarly, the average number of monthly page views has increased by a factor of six, from 297 in 2008 to 1,948 in 2011. These numbers show the interest in static KT terminology information. The international nature of the visitors is notable. Overall, most visitors are from the United States, Canada, the United Kingdom, and Australia – probably because of the vibrant KT research and activity in those regions. Considerable and increasing interest is shown in the field from such countries as India and China, which are not traditionally considered centres of KT activity. This suggests that KT research and development is likely poised for global growth. Growth in interest is also shown by the fact that most people come to the wiki through Google. Our definitions pages appear near the top of Google search results lists, indicating that a number of other sites have created links to the wiki, thus signaling interest in KT terminology.

Our results related to collaboration and dialogue were discouraging. WhatisKT wiki members who were not part of the project development have not edited content or interacted with each other. This lack of editing and commenting could be because of such factors as perceived difficulty in editing and lack of specific guidance for the visitors on how best to contribute. The relative lack of participation (*i.e.*, active editing) by the visitors is consistent with other web 2.0 applications: Contributors are only a very small portion of the total number of people who access the services for information [27]. According to Wikimedia's estimates, the larger Wikipedias (*e.g.*, English, German, French) have 0.02% to 0.03% of visitors actively contributing to content [28,29]. In general, authorship attribution related to wikis is important to only some contributors.

The WhatisKT wiki experience affords the following lessons for collaborative platforms: user registration is a barrier; user behavior can be observed but not explained by analysis of log tiles; and spurring collaboration using a platform such as a wiki requires further study and innovation. Registering as a member and editing or commenting within a wiki are more difficult than contributing to social media or commenting in an open forum. The exact nature and objectives of most of the visitors to the WhatisKT wiki are not yet known, but will be assessed in a 2013 survey of our members and users. These factors need to be ascertained to devise better strategies to foster collaboration using an online forum such as a wiki.

The average number of page views, time spent on site per visitor, and proportion of people who visited only one page can provide information about the online behavior of visitors to a website but not necessarily why they visited or the effects of their visits. The average number of pages viewed by the visitors to the wiki has remained between one and two throughout the period of study. We did see an increase in the amount of time spent on the whole site and per page viewed. Combined with a high proportion of people who only visited one page (bounce rate) of above 90% and approximately 40 seconds per visit to the site, this likely indicates that most of the users visit the wiki for information regarding a specific term and leave thereafter. The increase in the proportion of pages viewed for the terms with a proposed definition could show evidence of strong interest in improving our use of KT terms. An alternative explanation is that the increased use is based on the added presence of a link from the home page to these suggestions for definitions. Further study is needed to determine which explanation is warranted — both may be contributing to the very real increase in viewing.

We are planning a survey study to assess if visitors find the information useful. We hope that the wiki design and content would promote cross navigation between pages, but we do not see evidence of the wiki being used in such a way. Improving navigation between pages may increase the average time per visit. We are also finding new terms to add to the wiki over time and still believe that a better understanding of how we use KT terms is needed. New interactive websites are available that offer easier commenting and contributing facilities. We may need to modify our wiki, change our approach to encouraging contribution, or move it to another platform if more user participation is to be assured. Interactive websites have the advantage of ease of use, but they lack the meticulous versioning functionality of a wiki. Wikis were originally designed to be used ‘as a moderated list where anyone can be moderator and everything is archived’ [29]. Therefore, both versioning history and ease of use need to be considered in developing and maintaining collaborative knowledge platforms.

We are also planning to add content in relation to KT interventions — a list of interventions and the results of an international meeting to propose a simple framework for classifying KT interventions. This face-to-face meeting in Ottawa, sponsored by KT Canada on September 20–21, 2012, used the wiki as part of the planning. The meeting was designed to ‘strengthen KT interventions, and hence patient and population health, by clarifying terminology with the goal of improving evidence searching and synthesis, communication between research groups, disciplines, and countries and increasing the profile of KT in scientific and other arenas.’ Groups and individuals from many countries were invited. We plan to publish our recommendations and next steps. In addition, we will invite others to join our project through traditional publications, emails, and personal contact. The project will also include expansion and enhancement of the WhatisKT wiki to be more of a working tool for collaboration.

The outcome of using a wiki can be a ‘function of both the features of technology and whether and how that technology is appropriated by groups that use it; neither can be divorced from the other’ [30]. The 'wiki way' philosophy of sharing knowledge, inviting critique, presenting multiple points of view and actively endeavoring to influence and change others’ opinions must fit the culture of the group [20]. We need to use our own KT knowledge to move WhatisKT into being a useful and used product. One way to increase participation may be to organize a formal link between the members, after which joint research and collaborative consultations can be facilitated. In fact, face-to-face group cohesion has been reported to make wikis work well [15,20]. Multiple techniques are needed for successful collaboration groups, and we can learn from the work of others [31].Absence of a clear-cut social structure and incentive to collaborate have been suggested as possible reasons for a lack of engagement in collaborative technologies [30,31].

The ranking data showing which terms are accessed most often can be useful to determine where interest or confusion occurs in the domain. For example, because research utilization was the most often viewed term by a considerable margin, this might be a more acceptable umbrella term for cross discipline use.

We have shown increases in the number of non-interactive visits and the global spread of WhatisKT; we now need to harness KT knowledge and move to other methods of collaboration on KT terminology.

## Conclusion

People are interested in KT terms and definitions as shown by the large and consistent increase in the number of people who visit the WhatisKT wiki site. We also observed the movement of interest from initially Canada and then to Canada and the United States before expanding to many other countries, with large spikes in use from the United Kingdom and India. Although we sought to increase interaction with our site to enhance communication about choice of KT definitions, use was concentrated by those who sought KT definitions.

## Abbreviations

KT: Knowledge translation.

## Competing interests

The authors declare that they have no competing interests in the publication of this article.

## Authors’ contributions

RBH, KAM, NLW, HQ, and CL planned this project and provided input and guidance. KAM, CL, AK, HQ, and NLW planned and carried out the analyses and interpretation of the data. All authors approved of the final content of the paper.
